# THE SCIENCE OF BEHAVIOR CHANGE: MECHANISTIC RESEARCH IN PEOPLE WITH DEMENTIA AND THEIR CARE PARTNERS

**DOI:** 10.1093/geroni/igae098.0341

**Published:** 2024-12-31

**Authors:** Talea Cornelius

**Affiliations:** Columbia University Irving Medical Center, New York, New York, United States

## Abstract

**Background:**

Interventions to support health and well-being people with dementia (PWD) and their care partners (CPs) lack efficacy and potency. Understanding why these interventions do—or do not—produce desired change has potential to improve outcomes in PWD and CPs. The NIH-funded Science Of Behavior Change (SOBC) promotes a mechanism-focused approach to developing rigorous, transparent, scalable, and effective interventions.

**Methods:**

In the SOBC experimental medicine approach, researchers identify a mechanism hypothesized to underlie behavior change, measure the mechanism, influence the mechanism, and test whether change in the mechanism explains subsequent change in the outcome of interest.

**Results:**

The SOBC website hosts instructional materials, a measures repository, a checklist for designing, conducting, reporting, and evaluating mechanistic research across a range of study designs, specific aims templates, and more. International efforts are underway to rigorously link measures to potential mechanisms of action and encourage deep thinking about how to operationalize (measure) theoretical constructs. The challenges of moving from theory to study implementation are compounded in dyadic research (use of self v. partner report, analyzing individual reports v. dyad means, etc.).

**Conclusions:**

Study methodology must correspond to conceptual models, and valid measurement of mechanisms is essential. Casting a spotlight on the intricacies of dyadic research, particularly in PWD and CPs where one dyad member experiences progressive cognitive decline and the dyad must cope with these changes together, promotes more careful thinking around these important issues, and the SOBC method and resources can facilitate a more rigorous, transparent, and cumulative science.

